# Performance evaluation of the digital morphology analyser Sysmex DI-60 for white blood cell differentials in abnormal samples

**DOI:** 10.1038/s41598-024-65427-0

**Published:** 2024-06-21

**Authors:** Yan Zhao, Yingying Diao, Jun Zheng, Xinyao Li, Hong Luan

**Affiliations:** 1 National Clinical Research Center for Laboratory Medicine, Department of Laboratory Medicine, The First Hospital of China Medical University, Shenyang, 110001 Liaoning China; 2 Research Units of Medical Laboratory, Chinese Academy of Medical Sciences, Shenyang, 110001 Liaoning China

**Keywords:** Digital morphology analyzer, Manual counting, Sysmex DI-60, White blood cell differential, Optical imaging, Software, Assay systems, Cellular imaging, Classification and taxonomy, Image processing

## Abstract

Sysmex DI-60 enumerates and classifies leukocytes. Limited research has evaluated the performance of Sysmex DI-60 in abnormal samples, and most focused on leukopenic samples. We evaluate the efficacy of DI-60 in determining white blood cell (WBC) differentials in normal and abnormal samples in different WBC count. Peripheral blood smears (n = 166) were categorised into normal control and disease groups, and further divided into moderate and severe leucocytosis, mild leucocytosis, normal, mild leukopenia, and moderate and severe leukopenia groups based on WBC count. DI-60 preclassification and verification and manual counting results were assessed using Bland–Altman and Passing–Bablok regression analyses. The Kappa test compared the concordance in the abnormal cell detection between DI-60 and manual counting. DI-60 exhibited notable overall sensitivity and specificity for all cells, except basophils. The correlation between the DI-60 preclassification and manual counting was high for segmented neutrophils, band neutrophils, lymphocytes, and blasts, and improved for all cell classes after verification. The mean difference between DI-60 and manual counting for all cell classes was significantly high in moderate and severe leucocytosis (WBC > 30.0 × 10^9^/L) and moderate and severe leukopenia (WBC < 1.5 × 10^9^/L) groups. For blast cells, immature granulocytes, and atypical lymphocytes, the DI-60 verification results were similar to the manual counting results. Plasma cells showed poor agreement. In conclusion, DI-60 demonstrates consistent and reliable analysis of WBC differentials within the range of 1.5–30.0 × 10^9^. Manual counting was indispensable in examining moderate and severe leucocytosis samples, moderate and severe leukopenia samples, and in enumerating of monocytes and plasma cells.

## Introduction

The enumeration and classification of leucocytes (white blood cells, WBC) in peripheral blood provide crucial clinical information that facilitates the diagnosis and screening of haematological diseases^1^. Microscopy-based manual cell counting perceived as the gold standard for WBC differential assays, which warrants 200 cells on a Wright-stained peripheral blood smear (PBS) to be counted by two experienced cytologists^2^. However, the manual counting method is time-consuming and labour-intensive and necessitates the employment of extensively trained personnel^3–5^. Moreover, this approach exhibits a considerable degree of inter- and intra-observer variability^6^. Consequently, there is an increasing demand for developing digital microscope systems that can automatically analyse PBS morphology and improve laboratory efficiency.

The initial automated image analysis systems can be traced back to the 1960s, with notable examples, including CELLSCAN^7^, Hematrak^8^, and the Cydac Scanning Microscope System^9^. These systems possessed certain drawbacks, such as slow processing speed, constrained automation, and subpar accuracy, which consequently hindered their widespread employment^10,11^. The rapid advancements in digital imaging and information technology in recent years has affected the development and introduction of advanced automated digital microscopy systems to the laboratory. The CellaVision (AB, Lund, Sweden) and Sysmex DI-60 (Kobe, Japan) systems are the prevailing digital morphology (DM) analysers utilized in clinical laboratories^10,12^. Alternative DM analysers include EasyCell Assistant (Medica, Bedford, MA, USA), Vision Pro (West Medica, Perchtoldsdorf, Austria), and Nextslide (Nextslide Imaging, LLC, Cleveland, OH, USA)^10,13–15^.

The Sysmex DI-60 is an automated digital cell morphology analysis system that employs artificial neural network technology to locate, identify, and preclassify WBCs. It comprises a microscope equipped with two objectives (× 10 and × 100), relay tubes (× 1.0 and × 0.5), a camera, and a computer system housing the acquisition and classification software. It is the first integrated cell-locating image analysis system comprising an XN haematology analyser, slide maker, staining device, and DM analyser^16^. To date, numerous studies have evaluated the performance of DI-60 using clinical data^17–20^. Nevertheless, limited research has evaluated the performance of Sysmex DI-60 in abnormal samples, and most focused on leukopenic samples^5,19,21^. Our study covered both normal and abnormal samples (including haematological and non-haematological diseases), and comprised leucocytosis, normal in WBC number, and leukopenia. Therefore, this study aimed to assess the analytical performance of DI-60 in both leucocytosis, normal, as well as leukopenic samples and its ability to detect abnormal cells with reference WBC differentials assayed by manual counting.

## Materials and methods

### Study population

A total of 166 PBS specimens were collected from January to October 2023 from patients at the First Hospital of China Medical University. The WBC count in these specimens almost covers the entire clinically reportable range (0.11–271 × 10^9^/L). Blood samples were collected in K_2_-EDTA-containing vacuettes (Becton Dickinson and Company, Shanghai, China), and a Sysmex XN-9000 was used to conduct the CBCs for WBC differentials. The PBS slides were prepared and stained automatically using an SP-10 slide maker/stainer (Sysmex) and Wright's staining (Baso Company, Zhuhai, China), respectively. Subsequently, the stained specimens of healthy controls and patients were categorised into normal control (N = 50) and disease groups (N = 116, including 80 in haematological diseases group and 36 in non-haematological diseases group), and the disease group was further divided into five subgroups based on WBC counts: moderate and severe leucocytosis (> 30.0 × 10^9^/L, N = 28), mild leucocytosis (10.0–30.0 × 10^9^/L, N = 29), normal in number (4.0–10.0 × 10^9^/L, N = 23), mild leukopenia (1.5–4.0 × 10^9^/L, N = 19), moderate and severe leukopenia (< 1.5 × 10^9^/L, N = 17). In the hematological disease group, we selected 80 cases, including 46 acute leukemia, 13 lymphatic proliferative diseases, 3 anemia, 4 myeloproliferative neoplasms (MPN), 8 myelodysplastic syndrome (MDS), 6 MDS/MPN. The non-haematological diseases group consists of 21 infected disease patients, 4 autoimmune disease patients, and 11 other malignant tumor patients.

### WBC differentials obtained using DI-60 and manual counting

The slides prepared with SP-10 was loaded into the DI-60. Once the scanning start and end points on the slide had been established, the DI-60 initiated the scanning process in battlement-track mode, and was calibrated to count 200 cells per slide. The pre-classified cell images were displayed on a computer screen and subsequently verified by an expert. DI-60 was able to preclassify WBCs into 18 classes: leukocytes (segmented and banded neutrophils, lymphocytes, monocytes, eosinophils, basophils, blasts, promyelocytes, myelocytes, metamyelocytes, atypical lymphocytes, and plasma cells) and non-leukocytes (nucleated red blood cells, smudge cells, artifacts, giant platelets, platelet clumps, and unidentified cells)^17^. We investigated 11 classes of cells: band and segmented neutrophils, eosinophils, basophils, lymphocytes, monocytes, immature neutrophils, atypical lymphocytes, plasma cells, blast cells, and unidentified cells. Of these, immature neutrophils, atypical lymphocytes, plasma cells, and blast cells were considered abnormal in the WBC differentials.

Manual counting based on CLSI H20-A2 was independently performed by two experienced medical technologists with more than five years of experience in blood morphology examination^2^. The two experts counted 200 cells on each slide under a light microscope (Nikon Corporation, Japan), at 200 × magnification. The average number of cells counted by the two experts was obtained for evaluation.

### Statistical analysis

Data are presented as median (interquartile range, IQR) or arithmetic mean, number (percentage, %). The normality of the quantitative data distribution was assessed using the Shapiro–Wilk test. Sensitivity, specificity, positive predictive value (PPV), negative predictive value (NPV), Youden index, and area under the curve (AUC) of DI-60 preclassification based on verification were assessed using receiver operating characteristic curve analysis. Bland–Altman and Passing–Bablok regression analyses were performed to compare the DI-60 analysis and manual WBC differential counts. Pearson’s correlation coefficients (r) with 95% confidence interval (CI) were obtained and interpreted as follows: < 0.30, negligible; 0.30–0.50, low; 0.50–0.70, moderate; 0.70–0.90, high; 0.90–1.00, very high^22^. To compare the abnormal cell detection ability, the consistency between DI-60 and manual counting of abnormal cells in different groups was compared using the kappa test. Undetectable and discernible abnormal cells were defined as negative and positive, respectively. The Kappa result was interpreted as follows: values ≤ 0 as indicating no agreement, 0.01–0.20 as none to slight, 0.21–0.40 as fair, 0.41–0.60 as moderate, 0.61–0.80 as substantial, and 0.81–1.00 as almost perfect agreement^23^. Statistical analyses were performed using Microsoft Excel (version 2016; Microsoft Corporation, Redmond, WA, USA) and MedCalc Statistical Software (version 20; MedCalc Software, Ostend, Belgium); two-sided *P*-values less than 0.05 were considered statistically significant.

### Ethics approval

The present study has been reviewed and approved by Ethics Committee of the First Hospital of China Medical University, with the ethics approval number of [2023] N0.582. Informed consent was waived by the ethics committee of the First Hospital of China Medical University because the PBS slides were prepared using blood drawn from patients who, regardless of the study, were prescribed a complete blood count (CBC) and PBS examinations. This study does not require any other intervention measures. We confirm that all methods were carried out in accordance with relevant guidelines and regulations.

## Results

The detailed clinical features of the 166 patients, cells counted per slide by manual counting, DI-60 preclassification, and DI-60 verification are listed in Table 1. In manual counting, 200 cells (IQR, 200.0–200.0 cells) per slide were counted in 153 samples; less than 200 cells were counted in 13 samples. With the setting of 200 WBC counting, 23 samples in moderate to severe leukopenia group with less than 200 cells were counted by DI-60. There are 1920 undetermined cells in the DI-60 preclassification, including all kinds of white blood cells, degenerated cells, giant platelets, nucleated red blood cells, etc. Owing to the presence of non-leukocytes in the WBC differentials, such as degenerated cells, erythrocyte overlap, large platelets, nucleated red blood cells, and impurities in the dye solution, in DI-60 verification, only 18.0% of all specimens contained 200 cells.

**Table 1:** Sample characteristics.

	Total	Normal control group	Disease group (N = 116)				
	(N = 166)	(N = 50)	Moderate and severe leucocytosis group (N = 28)	Mild leucocytosis group (N = 29)	Nomal in number group (N = 23)	Mild leukopenia group (N = 19)	Moderate and severe leukopenia group (N = 17)
Age, median (IQR)	51 (34–63)	50 (35–61)	62 (48–68)	57 (25–66)	32 (6–60)	53 (29–59)	38 (34–49)
Male, N (%)	76 (45.8)	16 (32.0)	16 (57.1)	17 (58.6)	10 (43.5)	13 (68.4)	4 (23.5)
WBC counts (× 10^9^/L)	23.80 ± 6.87	5.75 ± 0.29	104.71 ± 24.26	18.12 ± 2.30	6.45 ± 0.67	2.44 ± 0.36	0.71 ± 0.23
Counted cells per slide by manual count, median (IQR)	200 (200,200)	200 (200,200)	200 (200,200)	200 (200,200)	200 (200,200)	200 (200,200)	200 (68,200)
Counted cells per slide by DI-60 preclassification, Median (IQR)	200 (200,200)	200 (200,200)	200 (200,200)	200 (200,200)	200 (200,200)	200 (199,200)	163 (85,200)
Counted cells per slide by DI-60 verification, median (IQR)	197 (190,199)	199 (198,200)	197 (192,199)	197 (194,198)	195 (191,198)	187 (135,196)	107 (56,170)
Abnormal cells							
Immature granulocytes	710	0	516	73	67	46	8
Atypical lymphocytes	733	3	4	349	334	37	6
Plasma cells	15	0	3	8	4	0	0
Blasts	3642	0	2140	281	606	426	189
Diagnosis, N (%)							
Haematological diseases	80 (69.0)	–	27 (96.4)	7 (24.1)	11 (47.8)	18 (94.7)	17 (100.0)
Non-haematological diseases	36 (31.0)	–	1 (3.6)	22 (75.9)	12 (52.1)	1 (5.3)	0 (0.0)

^a^Moderate and severe leucocytosis group, $WBC>$ 30.0 × 10^9^/L; mild leucocytosis group, WBC 10.0–30.0 × 10^9^/L; normal in number group, WBC 4.0–10.0 × 10^9^/L; mild leukopenia group, WBC 1.5–4.0 × 10^9^/L; moderate and severe leukopenia group, $WBC <$ 1.5 × 10^9^/L. ^b^Haematological diseases include acute leukemia, myelodysplastic syndromes, myeloproliferative neoplasm, anemia, lymphoproliferative disease, plasma cell leukemia; Non- haematological diseases include infections, autoimmune diseases, malignant solid tumors, etc.; the data is expressed as median (IQR), arithmetic mean or quantity (percentage, %). *IQR* interquartile range, *WBC* white blood cell.

To evaluate the performance of WBC preclassification by DI-60, we calculated the sensitivity, specificity, PPV, NPV, and efficiency for 166 samples. As shown in Table 2, the overall sensitivity was high for all cell classes, except immature granulocytes, ranging from 75.5 to 100.0%. The overall specificity, Youden index, and AUC were high for band and segmented neutrophils, eosinophils, lymphocytes, monocytes, immature granulocytes, atypical lymphocytes, plasma cells, and blasts (range 74.9–100.0%), and relatively low for basophils. The overall PPV was high for band and segmented neutrophils, eosinophils, lymphocytes, monocytes, immature granulocytes, and blasts (range 73.8–100.0%), and relatively low for plasma cells, atypical lymphocytes, and basophils (6.8%, 56.1%, 65.2%, respectively). The overall NPV was high for eosinophils, basophils, atypical lymphocytes, immature granulocytes, plasma cells, and blasts (range 83.9–100.0%), and relatively low for lymphocytes, monocytes, segmented neutrophils, and band neutrophils (27.3%, 33.3%, 45.0%, 58.0%, respectively). In subgroup analysis based on WBC counts, the sensitivity, specificity, PPV, NPV, Youden index, and AUC were similar to the overall results for all cell classes except for atypical lymphocytes. In addition, compared with the overall results, the Youden index and AUC of the haematopathy group were significantly reduced for all cell classes (Table S1).

**Table 2:** Performance of WBC differentials preclassification by DI-60 based on verification in total samples.

Cell class	Sensitivity (%, 95% CI)	Specificity (%, 95% CI)	Positive predictive value (%, 95% CI)	Negative predictive value (%, 95% CI)	Youden index	AUC (95% CI)
Band neutrophils	84.4 (77.2–90.1)	93.6 (78.6–99.2)	98.3 (93.7–99.5)	58.0 (48.0–67.4)	0.78	0.92 (0.86–0.95)
Segmented neutrophils	93.0 (87.8–96.5)	100.0 (66.4–100.0)	100.0	45.0 (31.6–59.1)	0.93	0.99 (0.96–1.00)
Eosinophils	92.9 (86.0–97.1)	88.1 (77.8–94.7)	92.0 (85.7–95.7)	89.4 (80.4–94.5)	0.81	0.93 (0.87–0.96)
Basophils	94.6 (87.9–98.2)	35.6 (24.7–47.7)	65.2 (61.1—69.1)	83.9 (67.7–92.8)	0.30	0.64 (0.57–0.72)
Lymphocytes	95.1 (90.6–97.9)	100.0 (29.2–100.0)	100.0	27.3 (16.0–42.4)	0.95	0.98 (0.96–1.00)
Monocytes	75.5 (67.7–82.2)	94.7 (74.0–99.9)	99.1 (94.3–99.9)	33.3 (27.0–40.4)	0.70	0.87 (0.81–0.92)
Immature granulocytes	68.9 (53.4–81.8)	90.9 (84.3–95.4)	73.8 (60.8–83.7)	88.7 (83.5–92.4)	0.60	0.85 (0.78–0.90)
Atypical lymphocytes	76.7(57.7–90.1)	86.7(79.9–92.0)	56.1 (44.3–67.2)	94.4 (89.8–97.0)	0.63	0.87 (0.81–0.92)
Plasma cells	100.0 (29.2–100.0)	74.9 (67.5–81.3)	6.8 (5.3–8.7)	100.0	0.75	0.90 (0.84–0.94)
Blasts	93.9 (83.1–98.7)	89.7 (82.8–94.6)	79.3 (69.1–86.8)	97.2 (92.1–99.1)	0.84	0.96 (0.92–0.99)

*CI* confidence interval, *WBC* white blood cells, *AUC* area under the curve.

Subsequently, the concordance of DI-60 preclassification and DI-60 verification with manual counting was compared for WBC differentials. The results depicted that the mean differences between DI-60 preclassification and manual counting ranged from − 4.36 to 1.93 in the total samples; between DI-60 verification and manual counting ranged from − 1.55 to 2.50. The mean differences between DI-60 and manual counting (− 6.30 to 2.57; − 2.21 to 2.91) in disease group were obviously higher than these in normal control group (− 2.51 to 1.69; − 2.02 to 2.86) (Table 3). For the subgroup analysis, the mean difference of the haematopathy group was higher than that of the non-haematological diseases group (− 9.37 to 3.27 vs − 4.21 to 1.44; − 1.60 to 16.63 vs − 2.52 to 3.57) (Table S2). The results of subgroup analysis based on WBC count showed that the mean difference between DI-60 and manual counting for all cell classes was significantly high in moderate and severe leucocytosis and moderate and severe leukopenia groups (Table S3). For the four classes of abnormal cells, the mean difference between DI-60 preclassification and manual counting of blasts was the highest at − 4.36 (95% CI − 6.42 to − 2.30) in the total samples, and between DI-60 verification and manual counting was − 1.55 (95% CI − 2.37 to − 0.73) (Table 3).

**Table 3:** Comparison of WBC differentials by DI-60 and manual count.

	Total, mean difference (%, 95% CI)		Normal control group, mean difference (%, 95% CI)		Disease group, mean difference (%, 95% CI)	
	Preclassification vs. manual count	Verification vs. manual count	Preclassification vs. manual count	Verification vs. manual count	Preclassification vs. manual count	Verification vs. manual count
Band neutrophils	− 0.91 (− 1.59 to − 0.22)	− 1.05 (− 1.61 to − 0.49)	0.63 (0.27 to 0.99)	− 0.06 (− 0.33 to 0.20)	− 1.57 (− 2.51 to − 0.62)	− 1.48 (− 2.26 to − 0.69)
Segmented neutrophils	0.86 (− 0.37 to 2.09)	2.50 (1.28 to 3.72)	1.69 (0.36 to 3.01)	2.86 (1.49 to 4.23)	0.50 (− 1.17 to 2.17)	2.35 (0.70 to 4.00)
Eosinophils	− 0.45 (− 1.08 to 0.17)	− 0.42 (− 1.04 to 0.20)	− 0.24 (− 0.47 to − 0.01)	− 0.14 (− 0.38 to 0.10)	− 0.55 (− 1.44 to 0.35)	− 0.58 (− 1.42 to 0.35)
Basophils	1.93 (1.29 to 2.57)	− 0.31 (0.13 to 0.48)	0.46 (0.23 to 0.68)	0.28 (0.12 to 0.44)	2.57 (1.67 to 3.46)	0.32 (0.08 to 0.56)
Lymphocytes	− 1.32 (− 3.47 to 0.84)	1.75 (0.41 to 3.09)	− 2.14 (− 3.47 to − 0.81)	− 0.94 (− 2.25 to 0.38)	− 0.96 (− 4.01 to 2.08)	2.91 (1.11 to 4.72)
Monocytes	− 3.03 (− 4.56 to − 1.50)	− 1.43 (− 2.70 to − 0.17)	− 2.51 (− 2.97 to − 2.04)	− 2.02 (− 2.49 to − 1.54)	− 3.25 (− 5.45 to − 1.06)	− 1.18 (− 2.99 to 0.62)
Immature granulocytes	0.11 (− 0.81 to 1.03)	− 0.60 (− 1.18 to − 0.02)	0.41 (0.24 to 0.58)	0	− 0.02 (− 1.34 to 1.29)	− 0.86 (− 1.68 to − 0.026)
Atypical lymphocytes	− 0.76 (− 1.32 to − 0.19)	− 0.13 (− 0.52 to 0.26)	0.17 (0.07 to 0.27)	0.02 (− 0.02 to 0.06)	− 1.16 (− 1.96 to − 0.36)	− 0.20 (− 0.76 to 0.36)
Plasma cells	− 0.72 (− 1.04 to 0.39)	− 0.06 (− 0.21 to 0.09)	0	0	0.68 (0.40 to 0.97)	0.02 (− 0.12 to 0.16)
Blasts	− 4.36 (− 6.42 to − 2.30)	− 1.55 (− 2.37 to − 0.73)	0.15 (0.06 to 0.24)	0	− 6.30 (− 9.20 to − 3.42)	− 2.21 (− 3.37 to − 1.06)

*CI* confidence interval, *WBC* white blood cell, *vs* versus.

**Figure 1:**
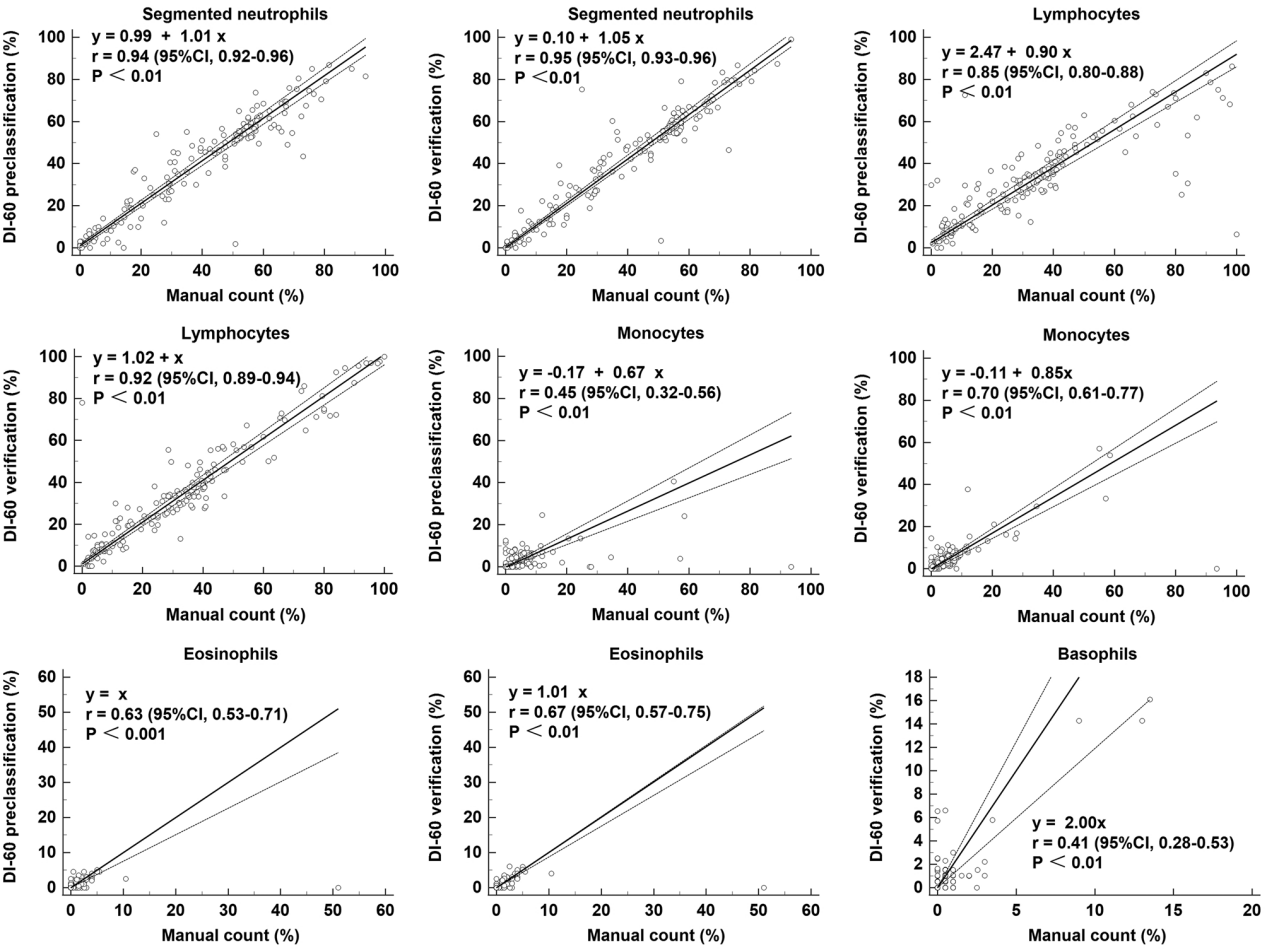
Comparison of WBC differentials between DI-60 and manual count in the five classes of WBC (n = 166). The data of basophils between DI-60 preclassification and manual count were not suitable for Passing-Bablok regression (r = 0.08, p = 0.33). Solid line, Passing-Bablok regression; dashed line, 95% CI line. *CI* confidence interval, *WBC* white blood cells.

Furthermore, as depicted in Fig. 1 and Fig. S1, the concordance of WBC differentials between DI-60 and manual counting was determined using Passing–Bablok regression analysis. DI-60 preclassification and manual count showed high correlations for segmented neutrophils (r = 0.94), band neutrophils (r = 0.79), lymphocytes (r = 0.85), and blasts (r = 0.80); moderate correlations for eosinophils (r = 0.63), immature granulocytes (r = 0.56), and atypical lymphocytes (r = 0.53); and low correlations for monocytes (r = 0.45). Data for basophils and plasma cells were not suitable for Passing–Bablok regression analysis. After verification, the correlation between DI-60 and manual counting improved for all cell classes (r = 0.41–0.98), and plasma cells showed negligible correlation.

To compare the ability to detect abnormal cells by DI-60, we calculated the kappa coefficient for the different WBC count groups (Table 4). In total samples, the DI-60 verification results of blasts present very good agreement with the manual counting results (Kappa coefficient = 0.96, 95% CI 0.91–1.00); Immature granulocytes and atypical lymphocytes demonstrated relatively good agreement (Kappa coefficient = 0.74, 95% CI 0.63–0.86; 0.78, 95% CI 0.66–0.91, respectively); Plasma cells showed poor agreement (Kappa coefficient = 0.27, 95% CI − 0.27–0.71). In subgroup analysis, there are significant differences between different types of abnormal cells, especially immature granulocytes, and atypical lymphocytes.

**Table 4:** Kappa test for detecting abnormal cells using DI-60 vs manual count.

Groups	DI-60 verification vs manual counting (Kappa, 95% CI)			
	Plasma cells	Immature granulocytes	Atypical lymphocytes	Blasts
Total	0.27 (− 0.17 to 0.71)	0.74 (0.63 to 0.86)	0.78 (0.66 to 0.91)	0.96 (0.91 to 1.00)
Diagnosis subgroups				
Haematopathy group	0.25 (− 0.20 to 0.70)	0.73 (0.58 to 0.87)	0.44 (0.12 to 0.77)	0.97 (0.87 to 1.00)
Non-haematological diseases group	NA	0.57 (0.29 to 0.85)	0.83 (0.64 to 1.00)	NA
WBC counting subgroups				
Moderate and severe leucocytosis	NA	0.70 (0.43 to 0.96)	− 0.06 (− 0.14 to 0.03)	0.87 (0.62 to 1.00)
Mild leucocytosis	1.00	0.59 (0.28 to 0.90)	0.85 (0.65 to 1.00)	0.84 (0.53 to 1.00)
Nomal in number	NA	0.65 (0.33 to 0.96)	0.74 (0.48 to 1.00)	1.00
Mild leukopenia	NA	0.51 (0.10 to 0.93)	0.83 (0.50 to 1.00)	0.89 (0.69 to 1.00)
Moderate and severe leukopenia	NA	1.00	0.64 (0.00 to 1.00)	1.00

*CI* confidence interval, *NA* not available, *vs* versus. Due to the absence of blasts or plasma cells in some subgroups, Kappa test (DI-60 verification vs manual counting) could not be performed in all subgroups.

## Discussion

Currently, an increasing number of automated digital morphological analysers are being used to replace manual counting in clinical laboratories, and several studies have evaluated the performance of DI-60^17,19,21^ and CellaVision DM96^24,25^. In this study, we evaluated the analytical performance of DI-60 using normal and abnormal specimens, including specimens from patients with haematopathy (acute leukaemia, myelodysplastic syndromes, myeloproliferative neoplasm, anaemia, lymphoproliferative disease) and non-haematological disease. In addition, we assessed the ability of DI-60 to detect abnormal cells (immature neutrophils, atypical lymphocytes, plasma cells, and blast cells) in different WBC count groups for the first time.

DI-60 preclassification and manual microscopy revealed nearly 200 cells in most samples, except for those in the moderate and severe leukopenia group. The performance of DI-60 in WBC preclassification was acceptable, with high sensitivity, specificity, Youden index, and AUC with verification for band neutrophils, segmented neutrophils, eosinophils, lymphocytes, monocytes, immature neutrophils, plasma cells, atypical lymphocytes, and blasts, whereas the AUC of basophils was lower than 0.80. This result is consistent with some previous research findings^20^, possibly due to the large particles in the cytoplasm of basophils, even covering the nucleus, making the morphology of the nucleus difficult to recognise by DI-60 preclassification^26^. Moreover, our study demonstrated low PPV (6.8%) and high NPV (100%) for plasma cells on WBC preclassification compared with verification. These findings imply that plasma cells may be underestimated or missed by DI-60 preclassification in clinical practice, and may also mistake some cells for plasma cells. WBC verification is required when numerous cells are pre-classified in PBS. The International Council for Standardization in Haematology recommends that trained and experienced haematology experts perform cell verification and morphological reviews^1^. The results of the present study support this hypothesis. Furthermore, our findings suggested the Youden index and AUC for all cell classes in haematopathy group were lower than the normal control group. This is possibly because haematopathy can lead to abnormal changes in the morphology of WBC or the appearance of abnormal cells, especially for patients with myelodysplastic syndrome, which increases the difficulty of DI-60 in recognising WBC.

In addition, in healthy and non-haematological patients, the mean differences of WBC differentials between DI60 and manual count were low (range − 4.21 to 3.57), the digital morphology analyzers DI60 can reduce the frequency of manual microscopy and enhance the overall efficiency of WBC differentials. Then, we found that the mean differences between DI-60 and manual counting tended to be larger in the moderate and severe leucocytosis (WBC > 30.0 × 10^9^/L) and moderate and severe leukopenia groups (WBC < 1.5 × 10^9^/L) than that in the other groups (Table S3), suggesting that the WBC count may affect the concordance between DI-60 and manual counting in WBC differentials. Our results are similar to those previously reported by Yoon et al.^15,21^. For leukopenia samples, Yoon et al. has reported that the WBC differentials of DI-60 did not show acceptable differences compared with manual counting^21^. Currently, only a few studies have compared DI-60 with manual counting in leucocytosis samples. We speculate that the following may be responsible for this discrepancy: first, an increase in WBC count in a single visual field and a decrease in counted visual fields may lead to deviations in WBC differentials, which can be improved by increasing the number of counted cells to 300 or 500 cells^25^; second, abnormal changes in the cellular morphology of WBC lead to mistaken identification of DI-60; and third, different staining methods can affect the analytical performance of DI-60 in accurately identifying WBCs^18^. Excessive WBC counts can lead to poor staining, which in turn makes DI-60 unable to recognise the cells.

A comparison between DI-60 preclassification and manual microscopy counting in the five classes of WBC showed moderate-to-high correlations, except for monocytes, which improved after verification. This was presumably attributable to the morphological complexity of monocytes; the morphology of the monocyte nuclei are twisted and folded^27^, increasing the difficulty of cell identification. Among the four types of abnormal cells, blasts displayed a substantially better correlation than that of immature granulocytes, plasma cells, and atypical lymphocytes in our study. This may have been attributable to the high numbers of blasts in our selected samples; DI-60 and lab technologists can easily identify blasts (large nuclear to cytoplasmic ratio, loose nuclear chromatin, prominent nucleoli), but it is difficult to identify immature granulocytes, plasma cells and atypical lymphocytes due to subjectivity. This viewpoint is similar to that reported in other studies^19–21^.

In the present study, manual microscope counting was used as the gold standard, and abnormal cells were detected using DI-60. As documented in Table 4, plasma cells were significantly different between DI-60 verification and manual slide review, and the kappa values of plasma cells was significantly lower than that of the other three types of cells (0.27, 95% CI − 0.17 to 0.71). This may be related to the low numbers of plasma cells observed in our study. Based on these findings, manual counting is irreplaceable for WBC differentiation, especially for plasma cells. In recent years, further improvements in the detection of abnormal cells have become the focus of digital morphological analysis^10,12,28^. Several studies have attempted to increase the number of cells counted or develop new deep learning algorithms to improve the ability to detect abnormal cells^29,30^. Consequently, the future development of digital artificial intelligence presents the prospect for the gradual improvement of the recognition ability of peripheral blood cells will gradually improve. Our study has certain limitations, as the number of plasma cells included in the study is insufficient, which may lead to biased results.

## Conclusions

In conclusion, DI-60 demonstrates consistent and reliable analysis of WBC differentials within the range of 1.5–30 × 10^9^/L and can replace light microscopy in routine practice to increase speed in blood films evaluation. However, manual counting was deemed indispensable in examining moderate and severe leucocytosis samples, moderate and severe leukopenia samples, and in enumerating of monocytes and plasma cells. Ultimately, mastering the analysis performance of DI-60 for different samples will preclude the omission of abnormal cells and potentially expedite laboratory workflow.

### Supplementary Information

Supplementary Information.

## Data Availability

All data used and analysed during the current study can be obtained from the corresponding author upon a reasonable request.
