# Supplementary material for: Performance evaluation of the digital morphology analyser Sysmex DI-60 for white blood cell differentials in abnormal samples
Source: Sci Rep. 2024 Jun 21;14:14344. doi: 10.1038/s41598-024-65427-0 (PMC11192923; doi:10.1038/s41598-024-65427-0)
Supplement: Supplementary file 1 — Supplementary Information. [file 41598_2024_65427_MOESM1_ESM.docx]

Supplementary materials

Table S1. Performance of WBC differentials preclassification by DI-60 on the basis of verification in different groups

| Cell Class | Sensitivity (%, 95% CI) | Specificity (%, 95% CI) | Positive Predictive Value | Negative Predictive Value (%, 95% CI) | Youden index | AUC (95% CI) |
| --- | --- | --- | --- | --- | --- | --- |
|  |  |  | (%, 95% CI) |  |  |  |
| Normal control group | | | | | | |
| Band neutrophils | 73.8 (58.0 - 86.1) | 87.5 (47.3 - 99.7) | 96.9 (83.1 - 99.5) | 38.9 (26.4 - 53.0) | 0.61 | 0.82 (0.68-0.91) |
| Segmented neutrophils | NA | NA | NA | NA | NA | NA |
| Eosinophils | 100.0 (92.0 - 100.0) | 100.0 (54.1 - 100.0) | 100.0 | 100.0 | 1.00 | 1.00 (0.93-1.00) |
| Basophils | 94.6 (81.8 - 99.3) | 92.3 (64.0 - 99.8) | 97.2 (84.2 - 99.6) | 85.7 (60.7 - 95.9) | 0.87 | 0.96 (0.86-1.00) |
| Lymphocytes | NA | NA | NA | NA | NA | NA |
| Monocytes | NA | NA | NA | NA | NA | NA |
| Immature granulocytes | NA | NA | NA | NA | NA | NA |
| Atypical lymphocytes | 100.0 (15.8 - 100.0) | 93.75 (82.8 - 98.7) | 40 (18.2 - 66.6) | 100.0 | 0.94 | 0.98 (0.90-1.00） |
| Plasma cells | NA | NA | NA | NA | NA | NA |
| Blasts | NA | NA | NA | NA | NA | NA |
| Haematopathy group | | | | | | |
| Band neutrophils | 84.2 (72.1 - 92.5) | 95.7 (78.1 - 99.9) | 98.0 (87.6 - 99.7) | 71.0 (57.2 - 81.8) | 0.80 | 0.92 (0.84 - 0.97) |
| Segmented neutrophils | 95.8 (88.1 - 99.1) | 88.9 (51.8 - 99.7) | 98.6 (91.5 - 99.8) | 72.7 (46.2 - 89.2) | 0.85 | 0.97 (0.91 - 1.00) |
| Eosinophils | 81.8 (64.5 - 93.0) | 87.2 (74.3 - 95.2) | 81.8 (67.7 - 90.6) | 87.2 (76.7 - 93.4) | 0.69 | 0.85 (0.75 - 0.92) |
| Basophils | 59.4 (40.6 - 76.3) | 68.8 (53.7 - 81.3) | 55.9 (43.2 - 67.8) | 71.7 (61.6 – 80.0) | 0.28 | 0.69 (0.57 - 0.78) |
| Lymphocytes | 93.5 (85.5 - 97.9) | 100.0 (29.2 - 100.0) | 100.0 | 37.5 (20.4 - 58.3) | 0.94 | 0.98 (0.92 - 1.00) |
| Monocytes | 83.6 (71.9 - 91.8) | 73.7 (48.8 - 90.9) | 91.1 (82.7 - 95.6) | 58.3 (42.8 - 72.4) | 0.57 | 0.82 (0.72 - 0.90) |
| Immature granulocytes | 81.8 (64.5 - 93.0) | 76.6 (62.0 - 87.7) | 71.1 (58.8 - 80.8) | 85.7 (74.1 - 92.6) | 0.58 | 0.80 (0.69 - 0.88) |
| Atypical lymphocytes | 62.5 (24.5 - 91.5) | 62.5 (50.3 - 73.6) | 15.6 (9.1 - 25.5) | 93.7 (85.8 - 97.4) | 0.25 | 0.61 (0.49 -0.71) |
| Plasma cells | NA | NA | NA | NA | NA | NA |
| Blasts | 81.6 (68.0 - 91.2) | 93.6 (78.6 - 99.2) | 95.2 (83.9 - 98.7) | 76.3 (63.9 - 85.4) | 0.75 | 0.93 (0.86 -0.98) |
| Non-haematological diseases group | | | | | | |
| Band neutrophils | NA | NA | NA | NA | NA | NA |
| Segmented neutrophils | NA | NA | NA | NA | NA | NA |
| Eosinophils | 95.5 (77.2 - 99.9) | 85.7 (57.2 - 98.2) | 91.3 (74.4 - 97.4) | 92.3 (63.6 - 98.8） | 0.81 | 0.95 (0.82 -0.99) |
| Basophils | 95.8 (78.9 - 99.9) | 33.3 (9.9 - 65.1) | 74.2 (65.6 - 81.2) | 80.0 (33.3 - 97.0) | 0.29 | 0.54 (0.37 -0.71) |
| Lymphocytes | NA | NA | NA | NA | NA | NA |
| Monocytes | NA | NA | NA | NA | NA | NA |
| Immature granulocytes | 66.7 (34.9 - 90.1) | 75.0 (53.3 - 90.2) | 57.1 (37.5 - 74.8) | 81.8 (66.2 - 91.2) | 0.42 | 0.77 (0.61 -0.90) |
| Atypical lymphocytes | 95.0 (75.1 - 99.9) | 100.0 (79.4 - 100.0) | 100.0 | 94.1 (70.3 - 99.1) | 0.95 | 0.97 (0.85 - 1.00) |
| Plasma cells | NA | NA | NA | NA | NA | NA |
| Blasts | NA | NA | NA | NA | NA | NA |
| Moderate and severe leucocytosis group | | | | | | |
| Band neutrophils | 86.4 (65.1 - 97.1) | 100.0 (54.1 -100.0) | 100.0 | 66.7 (41.1 - 85.1) | 0.86 | 0.93 (0.77 - 0.99) |
| Segmented neutrophils | 100.0 (86.8 - 100.0) | 100.0 (15.8 - 100.0) | 100.0 | 100.0 | 1.00 | 1.00 (0.88 - 1.00) |
| Eosinophils | 76.9 (46.2 -95.0) | 80.0 (51.9 - 95.7) | 76.9 (53.7 - 90.5) | 80.0 (59.0 - 91.8) | 0.57 | 0.81 (0.62 - 0.93) |
| Basophils | 75.0 (47.6 - 92.7) | 66.7 (34.9 - 90.1) | 75.0 (56.2 - 87.5) | 66.7 (43.9 - 83.6) | 0.42 | 0.75 (0.55 - 0.90) |
| Lymphocytes | NA | NA | NA | NA | NA | NA |
| Monocytes | 92.0 (74.0 - 99.0) | 66.7 (9.4 - 99.2) | 95.8 (82.2 - 99.1) | 50.0 (17.5 - 82.5) | 0.59 | 0.68 (0.48 - 0.84) |
| Immature granulocytes | 100.0 (79.4 - 100.0) | 33.3 (9.9 - 65.1) | 66.7 (57.3 - 74.9) | 100.0 | 0.33 | 0.65 (0.45 - 0.82) |
| Atypical lymphocytes | 100.0 (29.2 - 100.0) | 52.0 (31.3 - 72.2) | 20.0 (14.3 - 27.3) | 100.0 | 0.52 | 0.71 (0.51 - 0.86) |
| Plasma cells | NA | NA | NA | NA | NA | NA |
| Blasts | 87.0 (66.4 - 97.2) | 100.0 (47.8 - 100.0) | 100.0 | 62.5 (36.7 - 82.7) | 0.87 | 0.94 (0.79 - 1.00) |
| Mild leucocytosis group | | | | | | |
| Band neutrophils | NA | NA | NA | NA | NA | NA |
| Segmented neutrophils | NA | NA | NA | NA | NA | NA |
| Eosinophils | 94.7 (74.0 - 99.9) | 90.0 (55.5 - 99.7) | 94.7 (73.7 - 99.1) | 90.0 (56.9 - 98.4) | 0.85 | 0.95 (0.80 - 1.00) |
| Basophils | 68.4 (43.4 - 87.4) | 70.0 (34.8 - 93.3) | 81.2 (61.6 - 92.1) | 53.8 (34.9 - 71.7) | 0.38 | 0.72 (0.52 - 0.87) |
| Lymphocytes | NA | NA | NA | NA | NA | NA |
| Monocytes | NA | NA | NA | NA | NA | NA |
| Immature granulocytes | 70.0 (34.8 - 93.3) | 73.68 (48.8 - 90.9) | 58.3 (37.3 - 76.7) | 82.4 (63.6 - 92.6) | 0.44 | 0.77 (0.57 - 0.90) |
| Atypical lymphocytes | 88.9 (51.8 - 99.7) | 95.0 (75.1 - 99.9) | 88.9 (53.9 - 98.2) | 95.0 (74.9 - 99.2) | 0.84 | 0.93 (0.77 - 0.99) |
| Plasma cells | NA | NA | NA | NA | NA | NA |
| Blasts | 100.0 (29.2 - 100.0) | 100.0 (86.8 - 100.0） | 100.0 | 100.0 | 1.00 | 1.00 (0.88 - 1.00) |
| Normal in number group | | | | | | |
| Band neutrophils | 95.2 (76.2 - 99.9) | 100.0 (15.8 - 100.0) | 100.0 | 66.7 (22.8 - 93.1) | 0.95 | 0.98 (0.81 - 1.00) |
| Segmented neutrophils | NA | NA | NA | NA | NA | NA |
| Eosinophils | 81.8 (48.2 - 97.7) | 91.7 (61.5 - 99.8) | 90.0 (57.4 - 98.4) | 84.6 (60.8 - 95.1) | 0.73 | 0.89 (0.69 - 0.98) |
| Basophils | 92.3 (64.0 - 99.8) | 40.0 (12.2 - 73.8) | 66.7 (54.1 - 77.3) | 80.0 (34.4 - 96.8) | 0.32 | 0.53 (0.31 - 0.74) |
| Lymphocytes | NA | NA | NA | NA | NA | NA |
| Monocytes | NA | NA | NA | NA | NA | NA |
| Immature granulocytes | 80.0 (44.4 - 97.5) | 76.9 (46.2 - 95.0) | 72.7 (48.5 - 88.3) | 83.3 (58.3 - 94.7) | 0.57 | 0.81 (0.60 - 0.94) |
| Atypical lymphocytes | 90.9 (58.7 - 99.8) | 91.7 (61.5 - 99.8) | 90.9 (60.3 - 98.5) | 91.7 (62.7 - 98.6) | 0.83 | 0.96 (0.78 - 1.00) |
| Plasma cells | NA | NA | NA | NA | NA | NA |
| Blasts | 100.0 (66.4 - 100.0) | 92.9 (66.1 - 99.8) | 90.0 (57.7 - 98.3) | 100.0 | 0.93 | 0.99 (0.84 - 1.00) |
| Mild leukopenia group | | | | | | |
| Band neutrophils | 93.3 (68.1 - 99.8) | 100.0 (39.8 - 100.0) | 100.0 | 80.0 (37.6 - 96.4) | 0.93 | 0.99 (0.81 - 1.00) |
| Segmented neutrophils | NA | NA | NA | NA | NA | NA |
| Eosinophils | 80.0 (28.4 - 99.5) | 92.9 (66.1 - 99.8) | 80.0 (36.5 - 96.5) | 92.9 (69.1 - 98.7) | 0.73 | 0.84 (0.60 - 0.96) |
| Basophils | 100.0 (54.1 - 100.0) | 46.2 (19.2 - 74.9) | 46.2 (34.1 - 58.6) | 100.0 | 0.46 | 0.69 (0.44 - 0.88) |
| Lymphocytes | NA | NA | NA | NA | NA | NA |
| Monocytes | 81.8 (48.2 - 97.7) | 87.5 (47.3 - 99.7) | 90.0 (58.5 - 98.3) | 77.8 (49.3 - 92.6) | 0.69 | 0.88 (0.65 - 0.98) |
| Immature granulocytes | 66.7 (22.3 - 95.7) | 92.3 (64.0 - 99.8) | 80.0 (35.9 - 96.6) | 85.7 (65.7 - 95.0) | 0.59 | 0.81 (0.57 - 0.95) |
| Atypical lymphocytes | 66.7 (9.4 - 99.2) | 62.5 (35.4 - 84.8) | 25.0 (10.7 - 48.0) | 90.9 (65.9 - 98.1) | 0.29 | 0.67 (0.44 - 0.88) |
| Plasma cells | NA | NA | NA | NA | NA | NA |
| Blasts | 75.0 (34.9 - 96.8) | 100.0 (71.5 - 100.0) | 100.0 | 84.6 (62.4 - 94.8) | 0.75 | 0.95 (0.73 - 1.00) |
| Moderate and severe leukopenia group | | | | | | |
| Band neutrophils | 100.0 (59.0 - 100.0) | 90.0 (55.5 - 99.7) | 87.5 (52.2 - 97.8) | 100.0 | 0.90 | 0.93 (0.70 - 1.00) |
| Segmented neutrophils | 90.0 (55.5 - 99.7) | 85.7 (42.1 - 99.6) | 90.0 (59.2 - 98.2) | 85.7 (47.7 - 97.5) | 0.76 | 0.90 (0.66 - 0.99) |
| Eosinophils | 100.0 (59.0 - 100.0) | 80.0 (44.4 - 97.5) | 77.8 (50.3 - 92.4) | 100.0 | 0.80 | 0.96(0.74 - 1.00) |
| Basophils | 100.0 (15.8 - 100.0) | 53.3 (26.6 - 78.7) | 22.2 (14.3 - 32.9) | 100.0 | 0.53 | 0.62 (0.36 - 0.84) |
| Lymphocytes | 86.7 (59.5 - 98.3) | 100.0 (15.8 - 100.0) | 100.0 | 50.0 (21.6 - 78.4) | 0.87 | 0.93 (0.70 - 1.00) |
| Monocytes | 50.0 (18.7 - 81.3) | 85.7 (42.1 - 99.6) | 83.3 (42.4 - 97.1) | 54.5 (37.6 - 70.5) | 0.36 | 0.69 (0.42 - 0.88) |
| Immature granulocytes | 100.0 (29.2 - 100.0) | 85.7 (57.2 - 98.2) | 60.0 (29.4 - 84.4) | 100.0 | 0.86 | 0.93 (0.70 - 1.00) |
| Atypical lymphocytes | 100.0 (15.8 - 100.0) | 6.7 (0.2 - 31.9) | 12.5 (11.1 - 14.1) | 100.0 | 0.07 | 0.53 (0.28 - 0.77) |
| Plasma cells | NA | NA | NA | NA | NA | NA |
| Blasts | 100.0 (54.1 - 100.0) | 81.8 (48.2 - 97.7) | 75.0 (46.1 - 91.3) | 100.0 | 0.82 | 0.89 (0.65 - 1.00） |

Abbreviations: CI, confidence interval; NA, not available; WBC, white blood cells; AUC, area under the curve.

Table S2. Comparison of WBC differentials by DI-60 and manual count in haematopathy and non-haematological diseases groups

|  | Haematopathy group, mean difference (%, 95%CI) | | Non-haematological diseases group, mean difference (%, 95%CI) | |
| --- | --- | --- | --- | --- |
|  | Preclassification vs. Manual Count | Verification vs. Manual Count | Preclassification vs. Manual Count | Verification vs. Manual Count |
| Band neutrophils | -1.65 (-2.56 to -0.74) | -1.01 (-1.84 to -0.18) | -1.37 (-3.75 to 1.01) | -2.52 (-4.27 to -0.77) |
| Segmented neutrophils | 0.31 (-1.78 to 2.40) | 1.80 (-0.42 to 4.01) | 0.94 (-1.92 to 3.80) | 3.57 (1.45 to 5.70) |
| Eosinophils | -0.76 (-2.05 to 0.54) | -0.77 (-2.05 to 0.51) | -0.08 (-0.45 to 0.28) | -0.02 (-0.42 to 0.38) |
| Basophils | 3.27 (2.03 to 4.52) | 0.41 (0.067 to 0.75) | 1.00(0.38 to 1.61) | 0.12 (-0.10 to 0.34) |
| Lymphocytes | -2.05 (-6.38 to 2.29) | 3.87 (1.42 to 6.32) | 1.44 (-0.53 to 3.41) | 0.79 (-1.23 to 2.81) |
| Monocytes | -4.28 (-7.42 to -1.14) | -1.69 (-4.29 to 0.91) | -0.98 (-1.99 to 0.04) | -0.05 (-0.93 to 0.82) |
| Immature granulocytes | 0.36 (-1.35 to 2.07) | -0.82 (-1.72 to 0.08) | -0.87 (-2.84 to 1.10) | -0.94 (-2.78 to 0.91) |
| Atypical lymphocytes | 0.21 (-0.35 to 0.77) | 0.14(-0.16 to 0.44) | -4.21 (-6.19 to -2.24) | -0.94 (-2.65 to 0.77) |
| Plasma cells | 0.74 (0.36 to 1.12) | 0.03 (-0.17 to 0.23) | 0.55 (0.15 to 0.94) | 0 |
| Blasts | -9.37 (-13.40 to -5.34) | 16.63 (9.67 to 23.58) | -1.57 (-4.23 to 1.08) | -0.01 (-0.04 to 0.01) |

Abbreviations: CI, confidence interval; WBC, white blood cell.

Table S3. Comparison of WBC differentials by DI-60 and manual count in different subgroups based on WBC counts

|  | Moderate and severe leucocytosis group, mean difference (%, 95%CI) | | Mild leucocytosis group, mean difference (%, 95%CI) | | Nomal in number group, mean difference (%, 95%CI) | | Mild leukopenia group, mean difference (%, 95%CI) | | Moderate and severe leukopenia group, mean difference (%, 95%CI) | |
| --- | --- | --- | --- | --- | --- | --- | --- | --- | --- | --- |
|  | Preclassification vs. Manual Count | Verification vs. Manual Count | Preclassification vs. Manual Count | Verification vs. Manual Count | Preclassification vs. Manual Count | Verification vs. Manual Count | Preclassification vs. Manual Count | Verification vs. Manual Count | Preclassification vs. Manual Count | Verification vs. Manual Count |
| Band neutrophils | -1.91 (-3.36 to -0.46) | -0.59 (-1.78 to 0.61) | -0.88 (-3.57 to 1.81) | -2.62 (-4.54 to -0.71) | -2.64 (-5.08 to -0.20) | -1.91 (-4.16 to 0.34) | -1.87 (-3.84 to 0.09) | -0.80 (-2.55 to 0.94) | -0.37 (-2.22 to 1.48) | -1.16 (-3.08 to 0.75) |
| Segmented neutrophils | 4.59 (2.07 to 7.11) | 4.34 (1.71 to 6.98) | -1.29 (-4.89 to 2.32) | 2.20 (-0.41 to 4.82) | 1.57 (-0.65 to 3.79) | 2.59 (0.04 to 5.15) | -1.32 (-4.28 to 1.64) | 0.52 (1.67 to 2.71) | -2.58 (-10.44 to 5.28) | 1.01 (-8.40 to 10.42) |
| Eosinophils | -0.43 (-1.08 to 0.21) | -0.31 (-0.81 to 0.20) | 0.21 (-0.10 to 0.52) | 0.25 (-0.11 to 0.62) | -0.31 (-0.72 to 0.11) | -0.21 (-0.64 to 0.22) | -0.10 (-0.69 to 0.49) | -0.25 (-0.60 to 0.11) | -2.86 (-9.25 to 3.52) | -3.04 (-9.41 to 3.32) |
| Basophils | 2.17 (-0.039 to 4.38) | 0.36 (-0.15 to 0.86) | 1.09 (0.49 to 1.69) | 0.19 (-0.06 to 0.44) | 3.46 (0.43 to 6.48) | -0.03 (-0.44 to 0.39) | 3.91 (1.35 to 6.47) | 0.90 (-0.07 to 1.86) | 3.04 (1.94 to 4.13) | 0.30 (-0.54 to 1.14) |
| Lymphocytes | 5.08 (-1.77 to 11.93) | 3.27 (0.96 to 5.59) | -0.02 (-1.71 to 1.65) | 0.08 (-1.79 to 1.94) | 3.15 (-2.34 to 8.63) | 3.62 (0.17 to 7.07) | -6.98 (-14.67 to 0.70) | 1.72 (-1.67 to 5.11) | -11.34 (-24.50 to 1.81) | 7.54 (-2.81 to 17.90) |
| Monocytes | -2.33 (-6.33 to 1.67) | 0.43 (-1.29 to 2.14) | -1.28 (-2.75 to 0.19) | 0.16 (-1.85 to 2.17) | -1.40 (-4.32 to 1.51) | -0.23 (-1.83 to 1.37) | -2.97 (-6.19 to 0.24) | -0.91 (-2.53 to 0.70) | -10.98 (-23.83 to 1.88) | -7.71 (-19.50 to 4.08) |
| Immature granulocytes | -2.54 (-7.53 to 2.45) | -3.99 (-7.18 to -0.81) | 0.40 (-0.88 to 1.69) | 0.03 (-0.41 to 0.46) | 1.26 (-0.98 to 3.50) | 0.34 (-0.46 to 1.14) | 0.90 (-0.78 to 2.58) | 0.27 (-0.40 to 0.94) | 0.62 (-0.38 to 1.62) | -0.07 (-0.25 to 0.11) |
| Atypical lymphocytes | 1.07 (-0.01 to 2.14) | -0.015 (-0.18 to 0.15) | -1.77 (-3.45 to -0.09) | 0.16 (-1.16 to 1.48) | -4.02 (-6.69 to -1.34) | -1.79 (-3.88 to 0.31) | -0.71 (-2.19 to 0.77) | 0.76 (-0.34 to 1.86) | -0.45 (-1.53 to 0.64) | -0.03 (-0.75 to 0.70) |
| Plasma cells | 0.23 (-0.05 to 0.51) | -0.05 (-0.13 to 0.03) | 0.44 (-0.16 to 1.03) | -0.12 (-0.37 to 0.13) | 0.47 (0.03 to 0.91) | -0.087 (-0.27 to 0.09) | 1.34(0.26 to 2.42) | 0.31 (-0.34 to 0.95) | 1.39 (0.43 to 2.35) | 0.22 (-0.25 to 0.69) |
| Blasts | -14.09 (-22.20 to -5.98) | -3.45 (-6.28 to -0.61) | 2.86 (-2.15 to 7.86) | -0.33 (-1.31 to 0.65) | -4.32 (-8.43 to -0.21) | -2.30 (-5.56 to 0.95) | -6.26 (-12.67 to 0.15) | -2.51 (-5.34 to 0.32) | -2.63(-11.36 to 6.10) | -2.94 (-6.76 to 0.89) |

Abbreviations: CI, confidence interval; WBC, white blood cell.


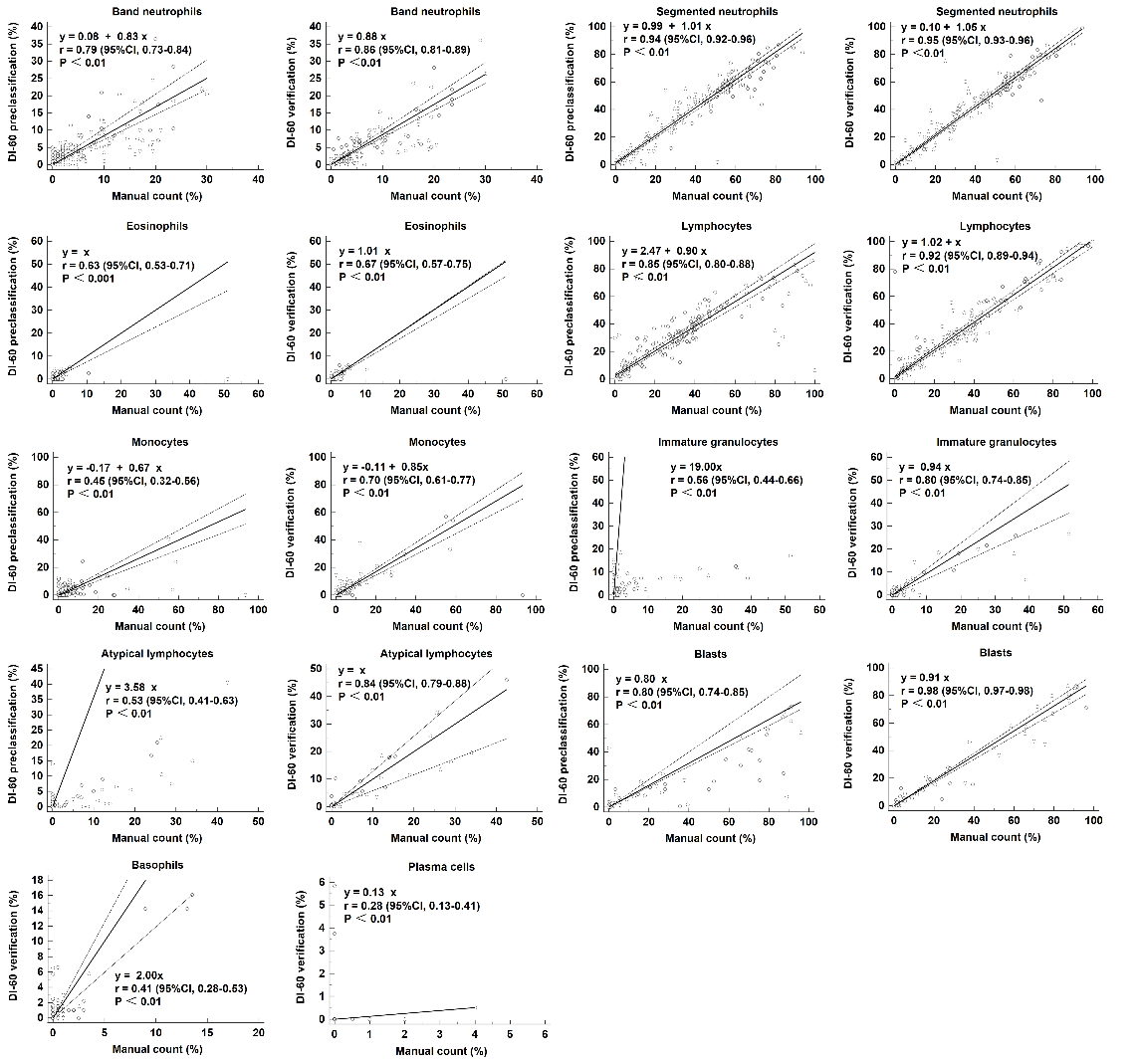


Figure S1. Comparison of WBC differentials between DI-60 and manual count (n = 166). The data of basophils and plasma cells between DI-60 preclassification and manual count were not suitable for Passing-Bablok regression (r = 0.08, p = 0.33; r = 0.06, p = 0.42). Solid line, Passing-Bablok regression; dashed line, 95% CI line. Abbreviations: CI, confidence interval; WBC, white blood cells.
